# Are Americans more successful at building intercultural relations than Japanese? A comparison and analysis of acculturation outcomes in Japan

**DOI:** 10.1186/2193-1801-3-716

**Published:** 2014-12-09

**Authors:** Adam Komisarof

**Affiliations:** Department of Economic Studies and Business Administration, Reitaku University, 2-1-1, Hikarigaoka, Kashiwa-shi, Chiba-ken, 277-8686 Japan

**Keywords:** Acculturation in Japan, Acculturation attitudes, Business intercultural communication, Nihonjinron

## Abstract

Various Western and Japanese sources in the literature have concluded that Japanese people, who live in a nation with comparatively less ethnocultural diversity than the U.S., lag behind Americans in their capabilities to develop positive intercultural relations. To test these assumptions, this study compared the quality of acculturation outcomes between Japanese and Americans in Japan. Japanese and American scores were calculated for five dependent measures used to operationalize quality of intercultural relations. Four dependent variables revealed no significant differences. For the variable of organizational investiture, Japanese had significantly higher scores, so data were analyzed to discern why.

**Electronic supplementary material:**

The online version of this article (doi: 10.1186/2193-1801-3-716) contains supplementary material, which is available to authorized users.

## Introduction

In modern nation-states, it is common to construct a coherent set of shared national traits that allow their members to function as “imagined communities,” or collectives of people who do not have face-to-face contact, yet by referencing these characteristics which they believe to be mutual, perceive themselves as members of the same group (Anderson 1991; Burgess 2010). In Japan, such self-ascribed traits frequently include cultural homogeneity and uniqueness, which imply a cultural distance from and difficulty communicating with the outside world (Befu 2001; Dale 1986; Goodman 2008; McVeigh 2004; Murphy-Shigematsu 2008). U.S. Americans, on the other hand, often view their nation as a product of continuous immigration (Steinberg 1981; Takaki 1993)—resulting in diversity which is a source of pride and strength (Furtado 2012). Their imagined community, by extension, consists of people regularly exposed to individuals from a variety of ethnocultural backgrounds; with that contact, they become reasonably proficient at developing positive intercultural relationships. These are assumptions that Japanese and Americans have not only about themselves, but also, to a certain extent, what they believe about each other. For instance, Japanese are often perceived by Americans and others from Western pluralistic democracies as being less exposed to ethnocultural diversity in the workplace and hence comparatively weaker at building positive intercultural relations as well as behind in accepting non-Japanese coworkers as core organizational members (Asai 2006; Kerr 2001; Kopp 1994; McConnell 2000; Murtagh 2005; Partridge 1987; Russell 1991).

But do these features of imagined communities stand up to scrutiny? Japan is now experiencing a demographic shift as greater numbers of non-Japanese workers are admitted to compensate for a projected labor shortage—one which is predicted in the face of a growing retiree population and a declining birth rate. Non-Japanese number about 2.04 million, or 1.6% of the entire population, which reflects a 50% jump from one decade earlier and nearly twice as many as in 1990 (Japanese Ministry of Justice 2013; Tabuchi 2011). Such changes have brought the concomitant challenge of how to integrate multicultural employees into their work organizations. So the aforementioned question about the veracity of imagined Japanese and American communal traits has important consequences as Japan’s population becomes more diverse and its corporations grow increasingly global: namely, by assessing such veracity, the current state of mutual acculturation outcomes between Japanese and American coworkers can be clarified, problematic acculturation dynamics identified, and better intercultural relations potentially facilitated.

According to Lueck and Wilson (2010), *acculturation* refers to “cultural changes resulting from primary contact between distinct ethnic groups” (p. 47), and it can involve *psychological acculturation*, or shifts in individual behaviors, attitudes, values, and identities (Berry et al. 1988; Smith Castro 2003). One way to test the accuracy of the aforementioned perceptions about imagined communities is to compare the quality of intercultural relations reported by Japanese and Americans in Japan to assess whether one group clearly has superior results—i.e., which group has more “successful” psychological acculturation outcomes at work. Therefore, the goals of this paper are to ascertain whether such differences exist—and if so, why.

Ideally, in order to compare American and Japanese proclivity in developing positive intercultural work relationships, a sample should include both Americans and Japanese working in Japan, as well as in the U.S.—where positions of sojourner and host are reversed. However, the focus in this study is consciously placed upon Japanese and Americans *in Japan* in order to test assumptions in the literature that Americans enjoy more successful outcomes of the psychological acculturation process—namely because this literature typically examines American-Japanese interactions in Japan. Therefore, this paper will test the robustness of such findings by taking a similar approach to the sources that produced them—i.e., sampling Americans and Japanese in Japan and confirming whether or not the literature’s conclusions about American and Japanese relational outcomes are supported.

It is also important to note that the acculturation of sojourners and long-term non-native residents to the host culture has inherent differences from the reciprocal process. While most acculturation scholars agree that acculturation is bidirectional (Sam 2006), the dominant group influences the acculturation-related attitudes and behaviors of non-dominant ethnocultural groups—exerting pressure to conform to their expectations for acculturation via interpersonal contact and social institutions (Berry 2006). Moreover, members of the host culture usually have broader choices as to whether and how they acculturate. In this study, it is understood that Americans (as an ethnoculturally non-dominant group in Japan) and Japanese people may experience the aforementioned differences in their mutual acculturation dynamics, so care is taken to select measures of acculturation outcomes for comparison which are relevant to both groups.

### Theoretical framework and hypotheses

#### Nihonjinron: *Its definition*, *inherent assumptions*, *and criticisms*

*Nihonjinron* is a genre of both academic and popular literature which attempts to define the identity of Japanese people, their cultural traits, and to establish the uniqueness of Japanese people, culture, and society (Sullivan and Schatz 2009). Such goals are often furthered through comparisons with other countries—most commonly the United States (Befu 2001). For example, while Japanese are usually portrayed as group-oriented and mutually interdependent, Americans are independent individualists. While Japanese ascribe to a hierarchical society, Americans prefer egalitarianism (Befu 2001; Dale 1986).

*Nihonjinron* also comprises an ideology or world view in which Japanese culture and identity are described with a set of qualities that distinguish Japanese from other national and ethnic groups—tracing those characteristics most commonly to geography, topography, rural community structure, or language (Befu 2001; Goodman 2008; Murphy-Shigematsu 2008). Central to *Nihonjinron* is the belief that land, race, language, and culture in Japan are coterminous; as Befu (2001) explained, Japanese people “inherited Japanese ‘blood’ from their forebears, . . . [and] have always lived on the Japanese archipelago” while “no other person speaks the language natively and practices [Japanese] culture” (p. 71). A variety of symbols are employed to evoke feelings of unity between Japanese and delineate ingroup boundaries, including Japanese ancestry, citizenship, linguistic fluency (Kidder 1992), literacy, and *joushiki* (“common sense”) in matters of daily comportment and judgment (which is developed during one’s primary socialization in Japanese society). Of course, there are Japanese who do not ascribe to *Nihonjinron*, but this ideology is widespread—promulgated in part by the *Nihonjinron* literature.

*Nihonjinron* engenders an exclusive national identity in that Japanese are depicted as a culturally homogenous, unique group whose features cannot be understood by non-Japanese (Befu 2001; Dale 1986; McVeigh 2004; Sakata 2009). As McVeigh (2004) inveighed, “Only Japanese can (or should) possess things Japanese” (p. 187). Americans are presumed unable to understand Japanese culture, become fluent or literate in the language, or practice a mainstream Japanese lifestyle (Cook 2006; Greer 2001). Views of Japanese culture and ethnicity embodied in *Nihonjinron* have been criticized by Western sociologists of Japan as “primordialist” (Goodman 2008), or as McVeigh (2004) warned, these “culturalist explanations and myths of uniqueness . . . [bolster] ethnic exclusivism, heightened ethnocultural self-consciousness, [and] racialized notions of identity” (p. 196). Consequently, some Western scholars have promulgated images of Japanese as insular, lagging behind in acceptance of diversity, and less adept at forming positive intercultural relations (Pacific Institute 1993). Dale (1986) argued that *Nihonjinron* conceptualizes Japan in terms of “feudal categories which social development in the West outgrew and transcended” (p. 44). More recently, McVeigh (2004) asserted, “Japan still seems behind the times” (p. 282) in terms of its rejection of cultural diversity within its borders and how its national identity is conceptualized around discourses embracing racialized exclusivity.

#### Cultural sources of Japanese weakness in intercultural communication: Arguments for and against

If Japanese do experience more negative acculturation outcomes than Americans, what might be their sources? According to McVeigh (2006), Japanese attribute their supposed difficulties in building positive intercultural relations to their culture’s uniqueness and incomprehensibility to outsiders—a conundrum frequently traced to the Japanese language; McVeigh (2004) asserted:

Many Japanese assume that their own language, being unique and exceptionally difficult, is beyond the capabilities of non-Japanese to learn. Consequently, they then assume that they themselves, being on the other side of an impenetrable linguistic wall, cannot learn a foreign language. (pp 244-245)

Likewise, Rivers (2011) reviewed ample literature positing that numerous Japanese view English proficiency as a threat to their Japanese identity; as a result, it is not uncommon to resist learning English and reject the possibilities it opens for communicating with the outside world.

But are Japanese people really uninterested in English language studies—and by extension, intercultural communication? Sakuragi (2008) contended, “Language education appears to occupy a far more important position in Japan than in the United States” (p. 82). Virtually all Japanese students study a foreign language, usually English, through six years of secondary education, and English ability is widely perceived as a key to scholastic achievement, college entrance, and career advancement. Moreover, many universities have programs in international/intercultural communication by popular demand. Therefore, as Sakuragi concluded, one could also argue that Japanese are actually more interested in other cultures than Americans.

Other cultural differences which purportedly hinder Japanese acculturation outcomes can be rooted, ironically, in concepts central to the field of intercultural relations. For instance, Nakane (1970, 1972) declared that Japanese struggle when communicating with non-Japanese because of “localism,” or the tendency for Japanese within the same collective to build a common nexus of insider knowledge and styles of expression which is not understood by outgroup members—even other Japanese. Such ingroup peculiarities, coupled with bonds so strong that they preclude developing relations with non-Japanese, impede effective intercultural communication and render Japanese society and culture difficult for foreigners to understand.

Similarly, Befu (2001) observed that the understanding of Japanese verbal and nonverbal messages, of which non-Japanese are widely presumed to be incapable, is enabled by a “body of unstated and implicit assumptions” (p. 39) that serves as the “context” in which such messages are to be deciphered. Making effective communication more difficult with Americans is the Japanese people’s sensitivity to their status relative to their speaking partner (which leads them to adjust their predicate endings and vocabulary choices accordingly) as well as Japanese conflict aversion—namely because Americans make comparatively few linguistic adjustments according to status and prefer forthright communication (Befu 2001; Nakane 1972).

Although *Nihonjinron* proponents do not usually employ the terms high/low context, individualism/collectivism, or large/small power distance, Japanese are characterized in this literature as being high context, large in power distance, and collectivist—resulting in a communication style that low context, small power distance, and individualist Americans cannot comprehend—much less employ themselves. Moreover, Japanese struggle to transcend the effects of being socialized in this communication style and its concomitant values, which distinguish yet isolate them—thus impeding positive relationships with non-Japanese. So *Nihonjinron* literature is quick to emphasize American-Japanese cultural differences and employ them to construct, legitimate, and reinforce ideas of Japanese uniqueness and the impenetrable intercultural communication barriers which invariably accompany it. Scholarship in the field of intercultural relations begins from the same point—i.e., acknowledging cross-cultural difference—yet it also stands on the assumption that tribulations in intercultural communication, including those between Japanese and Americans, can be ameliorated by cognitively grasping, affectively accepting, and behaviorally adjusting to the outgroup cultural differences which are at their base (Barnlund 1989; Gudykunst and Nishida 1994). In this view, which is also at the heart of culture learning theory (Masgoret and Ward 2006), cultural difference does not inherently cause communication problems—more important is whether we perceive it as an impenetrable barrier (which justifies rigid in- and outgroup distinctions) or attempt to acculturate and seek intercultural understanding.

In summary, though its conclusions have been contended, an ample body of literature written by both Americans and Japanese argues that Japanese are less proficient at building positive intercultural relations than Americans. Proponents of this view disagree as to whether such deficiencies among Japanese stem from ethnocentrism, a simple lack of exposure to diverse peoples, or a sense of cultural uniqueness which spawns an unbridgeable communication gap. Regardless, the clash between this view that Japanese are less capable intercultural communicators and its rebuttals from the intercultural relations field and anthropology beg this question to be empirically tested.

#### Choosing and operationalizing the variables

##### Independent variables

Subjects were categorized according to nationality: Japanese and Americans.

##### Dependent variables

Five dependent variables were chosen to operationalize the broader concept of quality of intercultural relations in the workplace based on the assumption that such quality includes both that of one’s relationships with cultural outgroup members *and* that of the work produced in intercultural work environments—namely because such work is a tangible product of intercultural interactions (Black 1988; Kealey 1989, 1996; Ward 1996). Moreover, these measures were deemed to be relevant relational outcomes for both Japanese and Americans—i.e., the ethnoculturally dominant and non-dominant groups—so long as they are engaged in regular intergroup contact in the workplace (which, as detailed in the section “Sample and survey characteristics,” was one of the requirements for participation in the study).

*Outgroup attitude* and *ingroup bias* were included as dependent variables since they have been emphasized as important acculturation outcomes in a variety of studies (Barrette et al. 2004; Bourhis et al. 1997; Montreuil and Bourhis 2001; Zagefka and Brown 2002). Outgroup attitude reflects how one rates cultural outgroup members on various work-related qualities, such as being hard-working or competent. Ingroup bias measures the difference between one’s ratings of cultural outgroup members and those of the same work-related qualities among cultural ingroup members, thus showing one’s predilection towards people with the same national background.

Acceptance among one’s cultural outgroup coworkers has far-reaching benefits for expatriates; for instance, it can positively impact job performance, as one can more readily gain assistance from others (Aycan 1997b). Moreover, acceptance usually comes with sustained host culture involvement, which has been shown to reduce sojourner stress and promote positive affect towards the host culture (Berry et al. 1987; Inoue and Ito 1993; Komisarof 2004; Sanchez and Fernandez 1993; Ward 1996). In organizational contexts, people feel accepted as insiders when they have opportunities to lead, be promoted, gain access to confidential insider knowledge, and participate in group decision-making (Harris 1995; Komisarof 2001; Lois 1999). Considering these indicators, Jones’ (1986) measure of *organizational investiture* was utilized to operationalize the degree of acceptance and support felt in intercultural work relationships.

The degree and depth of *social interaction* with cultural outgroup members impacts sojourner adjustment to the host culture (Furnham and Bochner 1986; Mendenhall and Oddou 1985; Palthe 2004; Tucker et al. 2004). Specifically, such contact presents opportunities to learn culture-specific skills and ameliorate sociocultural adaptation problems (Ward 1996). Aycan (1997a) further observed that contact with host national colleagues can reduce workplace conflict, teach organizational norms, and increase both job performance and commitment for expatriates.

Quality of work results was operationalized as *job effectiveness*—i.e., the subjective evaluation of one’s job performance (Ones and Viswesvaran 1997). The definition and measurement of success at work may change depending upon the type of sojourner, her job roles, and the skills which those roles demand (Kealey 1996), so job effectiveness was deemed an appropriate measure of work results since it is adaptable to the broad variety of sojourner types, job statuses, corporate divisions, companies, and industries included in this study.

#### Potential confounding variables

In order to assess how much variance in each dependent measure was accounted for by nationality and how much was actually shared with other sources, eighteen variables were tested as confounding variables.

**Figure 1:**
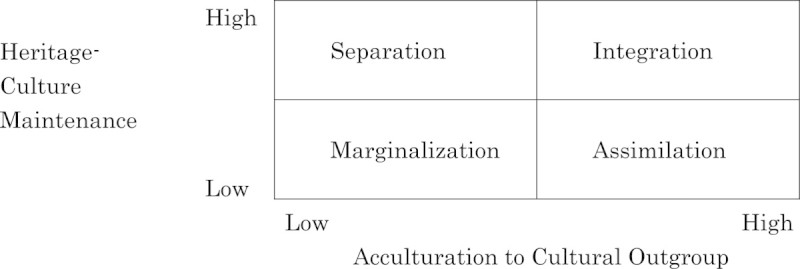
**The Berry framework of acculturation strategies.**

One potential confounding variable, acculturation strategies (Berry 2008; Berry et al. 2006), was proposed by Berry (1997a) to encompass both acculturation attitudes and their related behaviors. Such strategies are comprised of two independent, fundamental aspects which can be considered simultaneously: heritage cultural maintenance and intercultural contact (Berry and Sabatier 2010)—operationalized in this study as the extent that acculturating individuals strive to maintain their cultural attributes, as well as their degree of acculturation to their cultural outgroup (as depicted in Figure 1).

Individuals from either the dominant or nondominant ethnocultural group who embrace their own heritage cultural maintenance, but who oppose or do not see the importance of outgroup acculturation, adopt Separation strategies, while those who espouse acculturation to their outgroup, but reject or are unconcerned with their own cultural maintenance, choose Assimilation. Those who prefer both cultural maintenance and outgroup acculturation favor Integration, and those who desire neither are characterized by Marginalization. Acculturation strategies were tested as potential confounding variables because they relate specifically to people’s willingness to adapt to another ethnocultural group’s attitudes, values, and behaviors—thus impacting their daily intercultural communication dynamics and quality of intercultural relations.

**Figure 2:**
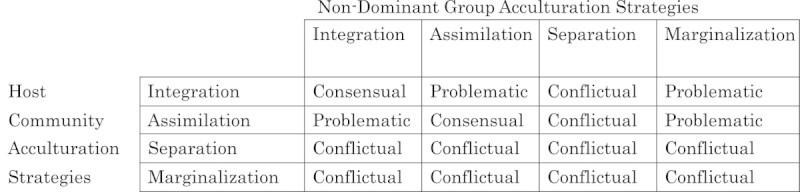
**The IAM as modified for this study.**

Bourhis and colleagues’ Interactive Acculturation Model (IAM) (Barrette et al., 2004; Bourhis and Dayan 2004; Bourhis et al. 1997; Montreuil and Bourhis 2001) was utilized to assess the degree of acculturation strategy compatibility between Japanese and American coworkers—another potential confounding variable. Based upon Berry’s acculturation strategy framework, different combinations of acculturation strategies between host society members and immigrants or long-term sojourners result in three types of alignments: Consensual (i.e., the most positive), Problematic, and Conflictual (the most negative). Each alignment yields distinct clusters of social-psychological acculturation outcomes in general societal contexts (Bourhis and Dayan; Bourhis et al.) or within work organizations (Bourhis and Barrette: Mergers and the vitality of organizations, submitted). Due to limitations of the scales used in the current study (i.e., their inability to identify the acculturation strategy of Individualism proposed in the IAM), a modified form of the IAM was utilized with the same four acculturation strategies as in Berry’s model, or 16 potential acculturation strategy combinations. For these 16 combinations, the IAM’s original outcomes of Consensual, Problematic, and Conflictual alignments were maintained as illustrated in Figure 2.

Another two measures included as potential confounding variables were *foreign language ability* and *social desirability bias*. Foreign language ability has been broadly noted in the literature as promoting successful acculturation outcomes (Kealey 1996), and measurements of social desirability bias identified subjects who were potential sources of unreliable data.

Many demographic variables were identified in previous research which could influence associations between the independent and dependent variables in this study and thus were tested as potential confounding variables: gender (Sinangil and Ones 2003); length of time on job assignment (Robie and Ryan 1996); years lived abroad (Aycan 1997a); previous intercultural work experience (Aycan 1997a); as well as marital status, intermarriage with cultural outgroup members, and demographic composition of neighborhood of residence (i.e., inhabited primarily by host culture members or other ethnocultural groups) (Kim 2001). The following demographic variables were also thought by the author to be potential confounding variables: company size, industry, location of corporate headquarters (i.e., Japan or America), ethnocultural minority status in one’s organization, whether or not one’s nationality matched that of corporate headquarters, native language, age, and highest level of education completed.

#### The hypotheses

Five hypotheses were tested to assess the relationship between national groups and acculturation outcomes—i.e., whether there were statistically significant differences between Japanese and Americans on the five dependent measures. In order to test the assumption that Americans are more adept at building positive intercultural relationships than Japanese, it was posited that Americans would have better acculturation outcomes, i.e., higher scores on all of the dependent variables except ingroup bias, which is a negative outcome. While all of the dependent measures related to quality of intercultural relations, each one was treated separately both theoretically and empirically in order to ascertain more precisely if and how Japanese and American acculturation outcomes differed. The hypotheses were:

- **H1**. Americans will be associated with more positive outgroup attitudes towards Japanese than Japanese will towards Americans.
- **H2**. Japanese will be associated with stronger ingroup bias than Americans.
- **H3**. Americans will be associated with a deeper degree of organizational investiture than Japanese.
- **H4**. Americans will be associated with greater social interaction with cultural outgroup coworkers than Japanese.
- **H5**. Americans will be associated with higher job effectiveness than Japanese.

## Methods

### Sample, survey, data, and procedures

#### Sample and survey characteristics

The population comprised Americans and Japanese working in organizations located in Japan and owned by either Japanese or American entities. Participants were limited to those who had lived in Japan and were employed in their current offices for at least four months, worked regularly with their respective cultural outgroup members (Japanese or Americans), and had jobs in corporate locations with at least two-thirds Japanese employees (thus making the national demographics more consistent at the offices surveyed). The survey was offered in both English and Japanese so subjects could respond in their native language. Moreover, the survey was translated from English to Japanese and back-translated, utilizing a target-language editor to ensure equivalency—as recommended by Brislin (1986).

#### Demographic data

Surveys were distributed to 327 people and 200 completed them (response rate =61.2%). Of these 200 surveys, six were excluded due to responses characterized by excessive social desirability. The sample (*N* =194) consisted of 97 Japanese and 97 American participants in 73 organizations. Seventeen different industries were represented: most frequently, education (29%), insurance (24%), and information technology (12%). The location of corporate headquarters was well-distributed between America (48.5%) and Japan (51.5%). Gender favored men (females =39.2% and males =60.8%), and age was concentrated most heavily in the 30s (43.3%), followed by the 40s (24.8%) and 50s (20.6%).

On average, participants had worked with their cultural outgroup for 10.5 years (either in Japan or elsewhere) and lived in a foreign country for 8.6 years. The mean for American subjects’ stays specifically in Japan was almost 12 years (142.8 months), with 67.1% of Americans having been there for over five years. Five years is significant because it is generally considered to be the maximum threshold for expatriates (Aycan and Kanungo, 1997). Most Americans (89.7%) had no time limit for their tenures in Japan stipulated in their contracts. Having lived in Japan for lengthy periods and the option of remaining permanently, they generally fit the profile of long-term or permanent residents—not temporary expatriates.

#### Sampling methods

Nonprobability methods of convenience and snowball sampling were utilized, which are appropriate when social sensitivities pose serious problems for locating and contacting potential respondents (Singleton et al. 1993). In recent years, strict procedures for handling personal information have become the norm in Japanese organizations, which have exacerbated the longstanding reluctance to cooperate with unsolicited research conducted by outsiders noted by Ogasawara (1998). Therefore, such nonprobability methods were considered most likely to produce compliance with the survey while recognizing that they would limit the generalizability of the findings.

#### Questionnaire measures

All variables were measured using seven-point Likert-type scales with the exception of the Social Desirability scale, which included the original five-point scale. Scales ranged from one (“strongly disagree”) to seven (“strongly agree”), or in the case of the Social Desirability scale, one (“strongly disagree”) to five (“strongly agree”).

#### Independent variable: Nationality

Participants were categorized as either Japanese or American.

#### *Dependent variables*: *Operationalization of quality of intercultural relations*

The dependent variables were measured with previously-validated scales—though some questions were modified within parameters acceptable in survey research to fit the population of this study. Montreuil and Bourhis’ (2001) scale was used to assess outgroup attitude: participants rated the extent that their cultural outgroup coworkers were hardworking, aggressive (reverse scored), competent, and friendly (Cronbach’s alpha =0.71). Ingroup bias was calculated with Zagefka and Brown’s (2002) method: the same four qualities as outgroup attitude were rated for one’s national cultural ingroup, a difference score was determined for each of the four items, and the values were combined into a single measure (with positive values representing ingroup bias) (α =0.68).

Questions from Jones’ (1986) Investiture scale (reproduced in Additional file 1) were modified so that the items no longer focused upon social support and acceptance from coworkers of any nationality, but rather from American or Japanese coworkers in one’s cultural outgroup. One item gauging group boundary permeability was added to clarify each subject’s degree of acceptance among cultural outgroup members through opportunities for organizational participation (e.g., chances to engage in group decision-making, adopt leadership roles, and be promoted). Cronbach’s alphas confirmed that this new item did not lower scale reliability, so it was scored with equal weight to Jones’ other items (α =0.63).

Tucker et al.’s (2004) Job Performance scale (Additional file 2) measured job effectiveness (α =0.81), and Tucker et al.’s Social Interaction scale (Additional file 3) assessed social interaction with cultural outgroup members (α =0.72).

#### Potential confounding variables

Participants rated their own acculturation strategies using the Vancouver Index of Acculturation (“VIA”) (Ryder et al. 2000), generating scores on separate scales for the Berry framework’s two dimensions: degree of heritage cultural maintenance and degree of acculturation to the cultural outgroup (e.g., for American subjects, “I am comfortable working with American/Japanese people”) for ten acculturation domains (e.g., values and behavioral norms) (α =0.76 for one’s heritage cultural maintenance and α =0.77 for acculturation to outgroup). The VIA was originally conceived and validated as a measure only of subjects’ own acculturation strategies, but by using reworded items about identical acculturation domains, participants in this study also rated the acculturation strategies of a coworker whom they felt was representative of their cultural outgroup members’ predominant attitudes and behaviors towards the subjects’ cultural ingroup (e.g., for American subjects, “[My Japanese coworker] is comfortable working with Japanese/American people”) (α =0.86 for cultural outgroup member’s heritage cultural maintenance and α =0.88 for their acculturation to subject’s culture).

According to the sample median, each VIA scale was subjected to a bipartite split in order to classify each subject and his cultural outgroup coworkers into one of the four acculturation strategies: Integration (high heritage culture and high outgroup acculturation scores), Assimilation (low/high scores, respectively), Separation (high/low scores), and Marginalization (low/low scores). Then, according to the scheme in Figure 2, the IAM alignment between each subject and his cultural outgroup coworkers’ acculturation strategies was categorized as Consensual, Problematic, or Conflictual.

Tucker et al.’s (2004) Communication scale appraised foreign language ability, i.e., communication competence in oral, written, and nonverbal mediums (e.g., for Japanese participants, respectively, “I can communicate my needs in an emergency situation by using English,” “I can read and understand most all of the English language newspaper,” and “I understand and use the non-verbal cues of American culture when I communicate with American people”) (α =0.92), and their Social Desirability scale checked for social desirability bias (e.g., “I'm always willing to admit it when I make a mistake” and “I have never intensely disliked anyone”) (α =0.70). In accordance with Tucker et al.’s guidelines, participants with Social Desirability scale scores of 25 to the maximum of 30 and a standard deviation of +/- 2 or greater for two or more of the other dependent measures had all of their data excluded (i.e., six subjects in total).

Table 1 lists the mean and standard deviation for the scales measuring each dependent measure and potential confounding variable.

**Table 1:** **Means and standard deviations for dependent measures and potential confounding variables**

Measure	***M***	***SD***
1. Outgroup attitude	19.09	3.15
2. Ingroup bias	-0.27	3.64
3. Social interaction	24.53	6.70
4. Investiture	27.38	5.63
5. Job effectiveness	42.21	7.18
6. VIA self: Heritage culture acculturation	54.75	7.36
7. VIA self: Outgroup culture acculturation	47.93	8.32
8. VIA other: Heritage culture acculturation	58.64	7.22
9. VIA other: Outgroup culture acculturation	42.76	10.72
10. Foreign language ability	38.67	9.93
11. Social desirability bias	19.78	3.86

#### Correlations for dependent measures

Correlations were run for the five dependent measures. Ingroup bias—a negative outcome—was predicted to inversely correlate with the other four dependent measures, while the other four dependent measures were expected to correlate positively. As indicated in Table 2, all twenty correlations between dependent variables were significant and ran in the expected directions.

**Table 2:** **Intercorrelations between dependent measures**

	1	2	3	4	5
1. Outgroup attitude	—				
2. Ingroup bias	-0.721^**^	—			
3. Social interaction	0.284^**^	-0.296^**^	—		
4. Investiture	0.386^**^	-0.162^*^	0.486^**^	—	
5. Job effectiveness	0.303^**^	-0.157^*^	0.410^**^	0.450^**^	—

^**^Correlation is significant at the 0.01 level (two-tailed).

^*^Correlation is significant at the 0.05 level (two-tailed).

#### Analysis of the data

Hypotheses 1—5 (i.e., “Americans will be associated with more positive outgroup attitudes/weaker ingroup bias/a deeper degree of organizational investiture/greater social interaction/higher job effectiveness than Japanese people”) were tested first by one-way ANOVAs to check whether there was an association between nationality and each of the dependent variables.

A series of tests was then executed to determine how much variance in each dependent variable was actually explained by confounds. First, using one-way ANOVAs for ordinal and interval variables and chi-squares for nominal variables, the following potential confounding variables were tested for significant associations with nationality: acculturation strategies, IAM acculturation strategy fit, foreign language ability, social desirability bias, and fourteen demographic measures which may influence acculturation outcomes (listed in the section “Potential confounding variables”). Acculturation strategies and acculturation strategy fit were tested by chi-squares at each level (i.e., each of Berry’s four strategies and the IAM’s three types) followed by an omnibus test.

Every potential confounding variable which was found to have a significant association with nationality from the univariate model was then tested for its confounding effect using linear regression. The confounding variables for each dependent variable were identified by testing a model in which each confounding variable was entered one at a time, with the first step being nationality and the second step nationality plus one potential confounding variable. When the effect of nationality was attenuated by the inclusion of any single confounding variable, multi-predictors (confounders) models were then utilized to show the collective confounding effects on each dependent measure: nationality was entered in the first step, and in the second, nationality plus all of the confounding variables. To assess how much effect of nationality from the univariate model (i.e., one-way ANOVAs) was accounted for by the confounding variables, squared semipartial correlations of nationality from the first and second steps in the multi-predictors (confounders) models were evaluated.

Finally, to gain insight into how the confounding variables associated with the dependent measures, in a series of post-hoc tests, one-way ANOVAs (for categorical variables) and correlations (for continuous variables) were utilized to ascertain the significance and directionality of each confounding variable’s association with its dependent variable.

## Results

### Hypotheses 1—5

The means and standard deviations for Japanese and Americans for the five dependent measures, as well as results from the one-way ANOVAs, are reported in Table 3. Significant univariate associations were found between nationality and four of the five dependent variables, with Americans having a higher group mean for outgroup attitude and job effectiveness, and Japanese having a higher group mean for ingroup bias and investiture. There were no national differences found for social interaction.

**Table 3:** **Main effects for nationality and dependent measures**

Dependent measure	Japanese ***M***	American ***M***	Japanese ***SD***	American ***SD***	F-ratio	***p***	***η***_***p***_^***2***^
Outgroup attitude	18.26	19.93	3.51	2.51	14.55^**^	<0.001	0.070
Ingroup bias	0.51	-1.05	3.97	3.10	9.26^**^	0.003	0.046
Social interaction	24.57	24.50	6.18	7.22	0.01	0.940	<0.001
Investiture	28.69	26.06	5.13	5.81	11.15^**^	0.001	0.055
Job effectiveness	40.61	43.80	7.28	6.74	10.06^**^	0.002	0.050

*Notes. η*_*p*_^*2*^ = partial eta-squared.

^*^p < .05. ^**^p < .01.

Next, the results are presented for the multi-predictors models (in Tables 4, 5, 6, and 7) testing how much the effect of nationality changed between the model with and without the confounding variables for each dependent measure. For social interaction, no confounding variables were tested since there was no significant difference between Americans and Japanese.

**Table 4:** **Collective confounding effects for outgroup attitude**

Variables	***B***	SE ***B***	***β***	***p***	sr	sr^2^
*Step 1* (*R*^*2*^ = 0.070 Δ*R*^*2*^ = 0.070)						
Nationality	1.67	0.44	.27^**^	<0.001	0.265	0.070
*Step 2* (*R*^*2*^ = 0.141 Δ*R*^*2*^ = 0.071)						
Nationality	1.08	0.61	.17	0.080	0.120	0.014
Gender	0.34	0.53	.05	0.525		
Age	0.08	0.03	.26^**^	0.005		
Internat assign	-0.06	0.04	-.16	0.125		
Years abroad	0.02	0.04	.06	0.585		
Assimilation	0.49	0.63	.06	0.436		
Separation	-0.30	0.65	-.04	0.645		
Marginalization	-0.72	0.57	-.11	0.210		

*Notes*. Internat assign = years worked with cultural outgroup. Integration was the reference for Assimilation, Separation, and Marginalization.

^*^p < .05. ^**^p < .01.

**Table 5:** **Collective confounding effects for ingroup bias**

Variables	***B***	SE ***B***	***β***	***p***	sr	sr^2^
*Step 1* (*R*^*2*^ = 0.070 Δ*R*^*2*^ = 0.070)						
Nationality	-1.56	0.51	-.21^**^	0.003	-0.214	0.046
*Step 2* (*R*^*2*^ = 0.079 Δ*R*^*2*^ = 0.008)						
Nationality	-0.90	0.68	-.12	0.187	-0.089	0.008
Gender	-1.22	0.59	-.16^*^	0.039		
Age	-0.11	0.03	-.30^**^	0.001		
Internat assign	0.04	0.04	.10	0.303		
Years abroad	-0.00	0.04	-.01	0.950		

*Notes*. Internat assign = years worked with cultural outgroup.

^*^p < .05. ^**^p < .01.

**Table 6:** **Collective confounding effects for investiture**

Variables	***B***	SE ***B***	***β***	***p***	sr	sr^2^
*Step 1* (*R*^*2*^ = 0.055 Δ*R*^*2*^ = 0.055)						
Nationality	-2.63	0.79	-.23^**^	0.001	-0.234	0.055
*Step 2* (*R*^*2*^ = 0.061 Δ*R*^*2*^ = 0.006)						
Nationality	-2.38	0.84	-.21^**^	0.005	-0.200	0.040
B degree	-0.39	1.13	-.04	0.729		
M degree	-1.22	1.19	-.10	0.304		

*Notes*. B degree = highest completed level of education is a bachelor’s degree; M degree = highest completed level of education is a master’s degree. For highest completed level of education, the reference was PhD for bachelor’s degree and master’s degree.

^*^p < .05. ^**^p < .01.

**Table 7:** **Collective confounding effects for job effectiveness**

Variables	***B***	SE ***B***	***β***	***p***	sr	sr^2^
*Step 1* (*R*^*2*^ = 0.050 Δ*R*^*2*^ = 0.050)						
Nationality	3.20	1.01	.22^**^	0.002	0.223	0.050
*Step 2* (*R*^*2*^ = 0.168 Δ*R*^*2*^ = 0.118)						
Nationality	-0.08	1.38	-.01	0.953	-0.004	<0.001^a^
Cultural outgroup spouse	-0.79	1.51	-.05	0.603		
Cultural ingroup spouse	-0.73	1.20	-.05	0.543		
Neighborhood	-3.54	1.32	-.20^**^	0.008		
Internat assign	0.12	0.08	.14	0.115		
Years abroad	0.14	0.08	.19	0.076		
Assimilation	-1.86	1.42	-.10	0.192		
Separation	-1.93	1.45	-.11	0.187		
Marginalization	-3.40	1.27	-.22^**^	0.008		

*Notes*. Neighborhood = lives in a predominantly expatriate or Japanese neighborhood; Internat assign = years worked with cultural outgroup. Having a spouse from another national culture and having a spouse from the same national culture were both referenced to unmarried status. Integration was the reference for Assimilation, Separation, and Marginalization.

^a^ =0.000016.

^*^p < .05. ^**^p < .01.

For outgroup attitude, when comparing nationality’s semipartial correlation on its own and with that of all of the confounding variables at once, calculations indicated that 79.5% of the variance associated with nationality was accounted for by the confounding variables. After this shared variance was removed from the effect, the national difference was no longer significant. In the model with all of the confounding variables, 82.8% of the variance associated with nationality was accounted for by the confounding variables, and once this shared variance was removed, the national difference for ingroup bias was no longer significant. In the model for investiture, 27.0% of the variance associated with nationality was shared by level of education, and there was a minimal confounding effect, as nationality was significant at *p* < .01 in both steps and the beta values were almost unchanged. For job effectiveness, 99.9% of the variance associated with nationality was accounted for by the confounding variables, and once this shared variance was removed, the effect of nationality was no longer significant.

Thus, for outgroup attitude, ingroup bias, and job effectiveness, the effect of nationality was shared by the confounding variables to the extent that a significant effect disappeared when confounding variables were taken into account. For investiture, the lone confounding variable, level of education, showed only a minimal confounding effect.

#### *Post*-*hoc tests of the significance and direction of associations between confounding variables and dependent measures*

In post-hoc tests, the confounding variables for each dependent variable were tested for significant associations with those dependent variables using one-way ANOVAs for categorical variables (results in Table 8) and correlations for continuous variables (see Table 9). When an association existed, then this relationship’s direction was noted.

**Table 8:** **Univariate associations for dependent measures and confounding variables**

Dependent measure	Confounding variable	F-ratio	***p***	Direction
Outgroup attitude	Gender	7.58^**^	0.006	Males > Females
	Acculturation strategy	2.76^*^	0.043	A > I > S > M
Ingroup bias	Gender	16.91^**^	<0.001	Females > Males
Investiture	Education level	2.07	0.129	
Job effectiveness	Inter spouse	2.11	0.124	
	Neighborhood	9.08^**^	0.003	E > J
	Acculturation strategy	3.50^*^	0.017	I > A > S > M

*Notes*. df = degrees of freedom; A = Assimilation; I = Integration; S = Separation; M = Marginalization; Inter spouse = whether spouse is from another national culture; Neighborhood = lives in a predominantly expatriate or Japanese neighborhood; E = predominantly expatriate neighborhood; J = predominantly Japanese neighborhood.

^*^p < .05. ^**^p < .01.

**Table 9:** **Correlations for dependent measures and confounding variables**

Dependent measure	Confounding variable	Pearson correlation	***p*** (2-tailed)
Outgroup attitude	Age	0.236^**^	0.001
	Internat assign	0.107	0.136
	Years abroad	0.220^**^	0.002
Ingroup bias	Age	-0.303^**^	<0.001
	Internat assign	-0.132	0.066
	Years abroad	-0.171^*^	0.017
Job effectiveness	Internat assign	0.234^**^	0.001
	Years abroad	0.297^**^	<0.0001

*Notes*. Internat assign = years worked with cultural outgroup.

^*^p < .05. ^**^p < .01.

## Discussion

### Status of the hypotheses

The status of the hypotheses can be summarized as follows. Hypothesis 1 (“Americans will be associated with more positive outgroup attitudes than Japanese”) and Hypothesis 2 (“Japanese will be associated with stronger ingroup bias than Americans”) were rejected: although American mean scores were significantly higher for outgroup attitude, and Japanese scores were greater than Americans’ for ingroup bias, these associations were artifacts of the confounding variables. For outgroup attitude, males scored significantly higher than females, and Assimilation was found to be the most positive acculturation strategy, followed by Integration, Separation, and Marginalization, respectively. Moreover, the greater the subject’s age and the number of years lived abroad, the better the outgroup attitude. For ingroup bias, the mean for women was significantly higher than men’s. Also, the greater the number of years worked with one’s cultural outgroup (a marginal association), age, and years lived abroad, the lower the ingroup bias.

Hypothesis 3 (“Americans will be associated with greater social interaction with cultural outgroup coworkers than Japanese”) was rejected as no such relationship was found. Hypothesis 4 (“Americans will be associated with a deeper degree of organizational investiture than Japanese”) was also rejected, as Japanese scores were significantly higher than those of Americans. Moreover, national difference was not fully accounted for by the lone confounding variable of level of education, so there was a minimal confounding effect. Hypothesis 5 (“Americans will be associated with higher job effectiveness than Japanese”) was rejected because the association was an artifact of the confounding variables. Job effectiveness was higher when subjects lived in an expatriate neighborhood (as opposed to one inhabited primarily by Japanese people) and the greater the number of years spent living abroad or working with cultural outgroup members. Integration acculturation strategies were most favorable, followed by Assimilation, Separation, and Marginalization, respectively.

In sum, rather than associations with nationality, differences in outgroup attitude, ingroup bias, and job effectiveness scores had more to do with the confounding variables, such as having experienced long sojourns abroad, ample time working with cultural outgroup members, and acculturation strategies indicating substantial acculturation to one’s cultural outgroup (i.e., Integration and Assimilation). Therefore, there was nothing unequivocally “better” about Americans’ quality of intercultural relations or acculturation outcomes—and in fact, Japanese people scored higher in terms of investiture.

### Analysis of frequent confounding variables

Five confounding variables, i.e., gender, years lived abroad, years worked with cultural outgroup members, age, and acculturation strategies, were found to act significantly in at least two of the independent-dependent variable associations and to correlate with the dependent variables in those associations. In this section, the results for each of these confounding variables are scrutinized in light of findings about the same variables in the literature, with the goal of gaining deeper insight into the relationship between these confounding variables and the acculturation outcomes of interest in this paper. Moreover, the implications of the recurring significance of these confounding variables are considered, and recommendations offered for future research.

In the current study, males had more positive outgroup attitudes and less ingroup bias than females, but plausible explanations as to why were not apparent. Sinangil and Ones (2003) indicated the opposite—i.e., women generally possess more highly-developed social and interpersonal skills than men, which help them as expatriates to establish social networks, integrate into foreign social environments, and be viewed as more cooperative by coworkers. While not the same as positive outgroup attitude or low ingroup bias, such skills seem congruous with them; consequently, further research is clearly necessary to clarify the impact of gender upon outgroup attitude and ingroup bias.

Years lived abroad acted as a confounding variable in the associations between nationality and outgroup attitude, ingroup bias, as well as job effectiveness. Its correlations with each of these dependent measures imply that living abroad is an important stimulus to improve these acculturation outcomes. Ward and Kennedy’s (1993) findings were complementary—i.e., length of residence in the host culture was a powerful predictor of sociocultural adaptation. Moreover, in the current study, working for extended periods of time with cultural outgroup members was associated with lower ingroup bias (marginally significant) and higher job effectiveness. This suggests that intercultural contact at work—whether made domestically or abroad—may facilitate positive attitude changes, effectiveness in intercultural work environments, and even sociocultural adaptation similarly to years lived abroad. These findings also provide indirect support for the contact hypothesis (Allport 1954; Amir 1998), though a more explicit test of this association is recommended for future research.

Age’s positive correlation with outgroup attitude and negative correlation with ingroup bias indicate that there may be a developmental aspect to these acculturation outcomes. Despite some useful studies in linking acculturation and development (Aycan 1997b), efforts to assess how ontogenetic development contributes to acculturation outcomes, particularly among adults, remain nascent (Berry 1997b; Berry et al. 2011; Schonpglug 1997). Oppedal (2006) argued that host culture competence is a developmental process among children and adolescents of immigrant parents. If host culture competence can be demonstrated to grow with age among adults, too, then further studies might also be able to establish a relationship between such competence and improving outgroup attitudes and weakening ingroup biases. Thus, while neither the current study nor the literature has untangled specifically how age interacts with outgroup attitude or ingroup bias among working adults, Oppedal’s work provides some direction for future research.

Scores for outgroup attitude and job effectiveness among subjects with Integration or Assimilation acculturation strategies were significantly higher than those among subjects adopting Separation or Marginalization. While ample research has concluded that Integration yields the most favorable acculturation outcomes (Smith Castro 2003), other scholars have contended that alternative acculturation strategies may be preferable—depending on the selection, definition, and assessment of acculturation outcomes as well as the broader social context in which acculturation is occurring (Birman 1998; Nguyen et al. 1999; Rudmin 2006; Ward and Rana-Deuba 1999). For example, Assimilation has been linked to enhanced sociocultural adjustment (Ward and Kennedy 1994). The findings in the current study support the conclusion that Assimilation *and* Integration associate with more positive acculturation outcomes than Separation or Marginalization and also underscore the need for more research which differentiates the social contexts in which positive acculturation outcomes are better supported by Assimilation and which by Integration.

In summary, the confounding variables of years lived abroad, years worked with cultural outgroup members, and acculturation strategies suggest that given the same length of residence abroad and time worked with cultural outgroup members, as well as acculturation strategies of either Integration or Assimilation, Japanese stand to develop acculturation outcomes which are comparable in quality to those among Americans. This is supported in the literature on cultural adjustment and acculturation—confuting the position of various Japanese *Nihonjinron* advocates and Western scholars of Japan that Japanese are somehow less adept than Americans at developing positive intercultural relations.

### Reasons for and caveats about higher investiture among Japanese

The finding that Japanese investiture was higher than that among Americans could be interpreted in two ways. First, it could mean that Japanese subjects actively improved their levels of investiture with American colleagues—namely, by developing robust American cultural competence through acculturation and English linguistic proficiency. But such evidence in the data is scant: Japanese acculturation strategies tended towards either Separation (23.7%) or Marginalization (41.2%), indicating only modest acculturation to American culture (a total of 64.9%), while Americans were much more likely to acculturate to Japan either as Integrationists (36.1%) or Assimilationists (29.9%) (a total of 66.0%). Moreover, a *t*-test (two-tailed) confirmed that there was no significant difference between the group means in Japanese (*M* =37.47, *SD* =8.44) and American (*M* =36.27, *SD* =11.24) foreign language ability *t*(193) =0.85, *p* = .399.

Another way of interpreting higher investiture among Japanese subjects is that Americans were perceived by Japanese as comparatively more accepting and supportive than Japanese were by Americans. Such acceptance and support, i.e., the defining characteristics of investiture, may have been related to the more thorough exposure that Americans generally had in their daily lives to Japanese people and culture than the Japanese subjects had to Americans, as well as American subjects’ deeper outgroup acculturation (in terms of prevalently adopting Integration or Assimilation strategies).

There were several demographic measures which indicated greater cultural outgroup exposure among Americans. Even though many Japanese participants had lived abroad at some point (*n* =62 out of 97, *M* =3.1 years), such sojourns were not as numerous and were far shorter on average than those for Americans (*n* =97 out of 97, *M* =14.2 years). Acculturation opportunities at work also diverged. One precondition for eligibility in this study was a workplace in which at least two-thirds of the employees were Japanese, so American subjects were constantly encountering Japanese people and having to make choices about whether or not as well as how to acculturate to Japan, while Japanese participants’ opportunities for intercultural contact at work were presumably less frequent and thorough (as there was a maximum of one-third Americans in their offices and often far fewer). Moreover, Americans had more acculturation opportunities outside of work: they were much more likely to have spouses from the outgroup culture (45.4% of the American sample had a Japanese spouse while none of the Japanese had an American one) and to live in neighborhoods where intercultural contact with the outgroup was highly probable (71.1% of the American sample lived in predominantly Japanese neighborhoods while 10.3% of the Japanese sample resided in mostly expatriate ones).

The interpretation that the Americans *in this study* were perceived as more accepting and supportive of Japanese does not indicate, though, that Japanese people *in general* are less accepting and supportive of Americans, as these results may have been influenced by self-selection bias in two ways. First, Americans with predominantly negative attitudes towards Japanese people and culture were likely to remove themselves from the pool of potential subjects by leaving Japan. Therefore, the American subjects in this study, who were largely long-term or permanent foreign residents of Japan (as detailed in the section “Demographic data”), were most probably there by choice and comparatively more content in their intercultural work relationships than Americans who had already repatriated or migrated elsewhere. In this sense, the Americans in this study were a select group within the broader American population. Moreover, there was a similar lack of equivalence between the Japanese and American subjects: the Americans were more likely to have *chosen* to work with Japanese (by virtue of living in Japan) than the Japanese subjects, who may have desired a job in an intercultural context, or who may have unexpectedly found themselves working with Americans after corporate takeovers, departmental transfers, or other unanticipated events. These two types of self-selection bias, operating at both the American intragroup level and on an intergroup level between Japanese and Americans, may have contributed to Japanese rating Americans better in terms of acceptance and support and thus Japanese having higher investiture scores.

Testing equivalent samples of Japan- and U.S.-based Japanese and Americans would help to determine whether results for investiture in the current study were the consequence of demographic differences commonly found between host culture members and long-term sojourners (e.g., among the latter, more years lived abroad or worked with cultural outgroup members) or actual national-level differences between Japanese and Americans that positively facilitate for themselves and/or negatively inhibit for others a sense of investiture (i.e., attitudes, values, and/or behaviors which support permeable organizational ingroup boundaries for members of one’s heritage culture vs. impermeable ones for cultural outgroup members). Even so, the results from this study suggest that there are few differences between Japanese and Americans *in Japan* in the quality of their acculturation outcomes (a context in which one would, due to self-selection bias, contrarily expect such outcomes to favor Americans). Therefore, with the exception of investiture, the findings of this study contradict both the *Nihonjinron* and Western literature which rate the quality of American intercultural relationships superior to those of Japanese.

### Limitations of this study and recommendations for future research

External validity, or the generalizability of the findings about this study’s sample to the broader populations of Japanese and Americans in Japan, is limited due to the nonprobability sampling methods employed. Moreover, the conclusions cannot be extended to Americans and Japanese living in other countries such as the United States. Ideally, future research will include not only a sampling of Americans and Japanese coworkers in Japan, but also of such coworkers in America, followed by a comparison of acculturation outcomes between these two sets of groups—i.e., full crossing of country of origin with country of residence. This would better enable discernment as to whether intergroup differences in investiture found in this study were a function of being long-term foreign residents of another country, or whether there are actual cross-cultural differences—regardless of country of residence—between Americans and Japanese in the relationship of nationality and investiture.

This research relied on self-report measures, which may be susceptible to same source (shared method) bias, but could be remedied in future research by supplementing such self-report measures with ratings by colleagues in one’s immediate work circle. Other limitations included: causality could not be fully addressed, and alternative explanations for the independent-dependent variable correlations could not be ruled out empirically.

### Contributions of this research

This study has contributed to the acculturation literature by highlighting the importance of differentiating when disparities in acculturation outcomes between two groups associate with national cultural group membership and when such correlations are actually artifacts of confounding variables. Consequently, future research should remain vigilant in distinguishing between cases when groups diverge in outcome variables due to culture-specific values, attitudes, and/or behaviors, and when such differences are primarily related to age, gender, opportunities afforded for outgroup contact, and other variables which have not been shown to vary across the cultural groups being examined.

Despite the aforementioned limitations, this study also contributed to the literature on Japanese intercultural relations. When comparing Japanese and Americans in a context which was very likely to reveal acculturation outcomes favoring Americans, i.e., long-term American residents of Japan and local Japanese, the only significant difference found was in investiture. Therefore, notions that Japanese are less proficient than Americans at building positive intercultural relationships have received little support in this study—suggesting that such “knowledge” would be better reconsidered.

## Conclusions

How can this study be utilized to improve American—Japanese intercultural relations as Japanese workplaces become more diverse? First, in the sample included in this study, it appears that there are few differences between Japanese and Americans in terms of the quality of their acculturation outcomes when they are given the same types of experiences—e.g., opportunities to work abroad or with cultural outgroup members domestically. This conclusion is a blow to Americans who see Japanese as behind in their capability to engender positive psychological acculturation outcomes and also to *Nihonjinron* adherents who believe Japanese to be so unique so as to render themselves compromised when communicating with the outside world.

However, the fact remains that many Americans in this study did not report the same level of investiture as their Japanese peers. Therefore, it is crucial that a sense of meaningful participation and belonging, rather than mere coexistence, be shared among the multicultural members of Japan’s work organizations, especially if the foreign labor force continues to expand. This can be accomplished by creating spaces in which non-Japanese employees feel accepted, supported, and can thrive professionally—conditions promulgated by nurturing inclusive acculturation strategies (i.e., Integration and Assimilation) and making more opportunities for well-managed, sustained intercultural contact—for example, as articulated in the contact hypothesis (Allport 1954; Amir 1998). Ultimately, these changes can enable Japan-based companies to more effectively compete for and retain elite foreign talent in the global marketplace and form more effective multicultural teams within their organizations.

## Electronic supplementary material

Additional file 1: Investiture scale (American/Japanese versions).(DOC 26 KB)

Additional file 2: Job Performance scale (American/Japanese versions).(DOC 26 KB)

Additional file 3: Social Interaction scale (American/Japanese versions).(DOC 25 KB)
