# Supplementary material for: Are Americans more successful at building intercultural relations than Japanese? A comparison and analysis of acculturation outcomes in Japan
Source: Springerplus. 2014 Dec 9;3:716. doi: 10.1186/2193-1801-3-716 (PMC4320228; doi:10.1186/2193-1801-3-716)
Supplement: Supplementary file 1 — Additional file 1: Investiture scale (American/Japanese versions).(DOC 26 KB) [file 40064_2014_1489_MOESM1_ESM.doc]

**Additional file 1: Investiture scale** (**American**/**Japanese Versions**)

The questions in this section ask about your opinions. Please answer each question as carefully as possible by circling *one* of the numbers in the scale which most accurately indicates your degree of agreement or disagreement with the statement provided. Use the following key, from 1 to 7, to help guide your answers:

Strongly Strongly

Disagree Neutral Agree

1 2 3 4 5 6 7

1. I have been made to feel by almost all of my Japanese/American colleagues that my skills and abilities are very important in this organization.

2. Almost all of my Japanese/American colleagues have been supportive of me.

3. I have had to change my attitudes and values to be accepted by my Japanese/American coworkers in this organization. (Reverse scored)

4. My Japanese/American colleagues have gone out of their way to help me adjust to this organization.

5. I feel that Japanese/American organizational members have held me at a distance. (Reverse scored)

6. In my opinion, we Americans/Japanese have the possibility to participate completely in daily corporate life (for example, when appropriate, engage in group decision-making, take leadership roles, as well as receive a fair chance at promotions).
