# Supplementary material for: Are Americans more successful at building intercultural relations than Japanese? A comparison and analysis of acculturation outcomes in Japan
Source: Springerplus. 2014 Dec 9;3:716. doi: 10.1186/2193-1801-3-716 (PMC4320228; doi:10.1186/2193-1801-3-716)
Supplement: Supplementary file 2 — Additional file 2: Job Performance scale (American/Japanese versions).(DOC 26 KB) [file 40064_2014_1489_MOESM2_ESM.doc]

**Additional file 2: Job Performance scale** (**American**/**Japanese Versions**)

The instructions and Likert-type scale were the same as in Appendix A.

1. My job here is not interesting. (Reverse scored)

2. I have developed a network of personal relationships with Japanese/American people who help me to succeed with my work.

3. I work well with Japanese/American people in doing my job here.

4. I am well-adjusted to this workplace.

5. I do not enjoy my job here. (Reverse scored)

6. I am effective in my job here.

7. My company would say that I am doing well on my job duties.

8. When important, I have been able to share my knowledge and expertise with Japanese/American employees.
