# Supplementary material for: Are Americans more successful at building intercultural relations than Japanese? A comparison and analysis of acculturation outcomes in Japan
Source: Springerplus. 2014 Dec 9;3:716. doi: 10.1186/2193-1801-3-716 (PMC4320228; doi:10.1186/2193-1801-3-716)
Supplement: Supplementary file 3 — Additional file 3: Social Interaction scale (American/Japanese versions).(DOC 25 KB) [file 40064_2014_1489_MOESM3_ESM.doc]

**Additional file 3: Social Interaction scale** (**American**/**Japanese versions**)

The instructions and Likert-type scale were the same as in Appendix A.

1. I frequently get together socially with Japanese/American coworkers.

2. I have made friends with Japanese/American coworkers and spend time with them outside of the workplace in the evenings and on weekends.

3. I have difficulty making friends with my Japanese/American coworkers. (Reverse scored)

4. I spend most of my free time with expatriate coworkers from my own country or other countries as opposed to Japanese coworkers. (Japanese version: I spend most of my free time with Japanese coworkers as opposed to American coworkers.) (Reverse scored)

5. I prefer to socialize with other expatriate/Japanese coworkers, as opposed to Japanese/American coworkers. (Reverse scored)

6. I feel socially isolated among my Japanese/American coworkers. (Reverse scored)
